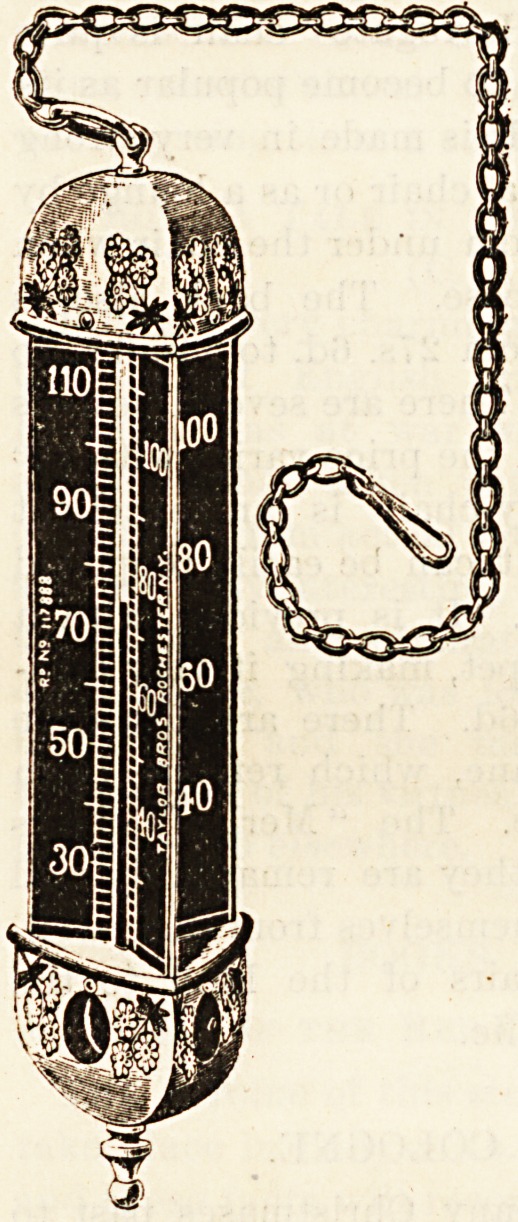# "The Hospital" Nursing Mirror

**Published:** 1900-12-15

**Authors:** 


### "The Hospital" Nursing Mirror.

Being the Nursing Section of "The Hospital."

[Contributions for this Section of "The Hospital" should be addressed to the Editor, "The Hospital" Nursing Mirror, 28 & 29 Southampton Street, Strand, London, W.C.]

### Notes on News from the Nursing World.

### PRESENTATION BY THE PRINCESS OF WALES ON HER HOSPITAL SHIP.

As on former occasions the Princess of Wales visited Southampton to inspect her hospital ship on its arrival for the third time from South Africa, and to inquire personally as to the health of the patients. The civil surgeon gave Her Royal Highness, who spent an hour and a quarter on board and conversed with each patient, particulars of the worst cases; and she herself saw one sufferer, who was wounded in eight places, carried on an ambulance chair out of the ship, when he was transferred, with over twenty other patients, to the train *en route* for the Gables Hospital at Surbiton. An unexpected, but most interesting, ceremony took place on the vessel before the Princess of Wales left. To their great delight, Her Royal Highness presented each of the four nursing sisters, Misses Chadwick, Brebner, Hogarth, and Spooner, with a souvenir brooch. The brooch is a charming decoration, consisting of a white enamel cross surmounted by a gold crown, the front of the cross bearing the initial "A" in gold. As the Princess of Wales has personally spent £8,000 on the fittings of the vessel which bears her name, it is incredible that the ship will be permitted to go out of commission, though she is not to return again to South Africa.

### THE PAY OF ARMY NURSES.

The members of the Army Nursing Service Reserve in South Africa are receiving more substantial remuneration than they anticipated. In fact, the allowances are so good that, according to one of their number who speaks from personal experience, they are all saving money. The pay, gratuity, and allowances of a superintendent amount to £226 a year, out of which £55 has to be paid for food, fuel, servants, and washing; while that of an ordinary nursing sister is £206. We are very glad to learn that the apprehensions of those nurses who expected that pecuniarily they would be worse off in South Africa than they were at home, have not been realised. It is only fair that the valuable services they render in positions of hardship and peril should be paid for on a liberal scale, and the War Office authorities are to be congratulated upon the manner in which they have recognised this obligation.

### "B.P." AND THE NURSES.

The following story is told by a correspondent of the *Daily Telegraph*. General Baden-Powell applied to a P.M.O. for volunteers among his nurses. After some days the medico arrived at headquarters. "Look here," he said, "a lot of my young women would like to join, but they want, first of all, to know what the kit is to be like." B.-P. rapidly created a picturesque study of a pretty girl, made still more pretty by a khaki blouse and a B.-P. hat, with a green puggaree and a jay's wing. The nurses were delighted, and the P.M.O. returned the next day. "You can take your pick," he said; "they're all crazy to join." The suggestion is, of course, that the pretty kit was the cause of the "craziness"; but we fancy that there is not a single member of the Army nursing service in South Africa who would not, apart from the question of uniform, have volunteered to nurse at the wish of the defender of Mafeking.

### AN UNFOUNDED COMPLAINT FROM NEW ZEALAND.

Commenting last week on the statement of Miss Emily Nicol, secretary of the Red Cross Brigade, Auckland, New Zealand, who accused the military authorities of refusing to employ colonial nurses, and especially New Zealand nurses, in South Africa, we suggested that the particular, as well as the general, assertion might be incorrect. We have since learned that, as a matter of fact, two nurses from New Zealand were, at any rate, accepted by the War Office. They were subsequently patients in one of the hospitals at Bloemfontein, as were also nurses from Canada, New South Wales, Kimberley, and Johannesburg, forming together a most cosmopolitan collection of "sick sisters." "The offer of New Zealand" to send nurses to tend the sick and wounded soldiers was therefore neither ignored nor declined.

### DECORATIONS FOR PLAGUE NURSES.

The decorations of the Order of St. John of Jerusalem to the nurses who earned them in connection with plague work were presented last month by Lady Northcote, wife of the Governor, in the Town Hall, Bombay. Lady Northcote thanked the Committee for doing her the honour of allowing her to give the badges to the nurses, and thus "affording her the opportunity of showing her deep appreciation of the splendid work done by these ladies."

### OUR CLOTHING DISTRIBUTION.

A final word to the kind friends who assist us in our Christmas clothing distribution. Monday next, December 17th, is the last day for sending in parcels. They should be addressed to the Editor of The Hospital, 28 and 29 Southampton Street, Strand, W.C., and the name and address of the sender should be enclosed in each parcel. We hope to be able this year to despatch a gift of warm and useful articles to more than the usual number of institutions where they are so warmly welcomed and so keenly appreciated. We have to thank the following readers for contributions received: Mrs. Reginald W. Lovelock, 10 Sydenham Avenue, Sefton Park, Liverpool; Miss H. M. Grafton, Wessington Court, Woolhope, Hereford; Policy Holder 1,080; Miss S. Macdonald, 24 St. James's Square, Bath; Miss K. Prior, 6 Percival Terrace, Kemp Town, Brighton; Nurse Cecil, Streatham; Nurse Groom, 123 New Bond Street, W.; Nurse Rodgers, 82 Station Road, Epsom; Nurse Willson, Westerham; Miss Henslowe, Preston Vicarage, Weymouth; Nurse Florence D. Lewis, The Hall, Caton, Lancaster; Nurse Mary Healy, Downing House, Cliftonville Avenue, Margate; and "Anonymous," a box containing stockings, &c.

### CHRISTMAS AT ST. MARY'S HOSPITAL.

The decorations will be quite simple at St. Mary's Hospital—just enough holly and ivy to gladden the eyes and cheer the hearts of the homesick sufferers who will be separated, perhaps for the first time, from home and loved ones. No Chinese lanterns will be lighted. There will be plenty of plants and flowers on the ward tables, and every effort will be made on that day to make each ward as home-like as possible. Any patients seriously ill will be allowed to have one or two of their nearest relations with them all day. On Christmas Day and Boxing Day smoking will be allowed in the men's wards when the condition of the patients admits of it. Pianos will be placed in certain of the wards on Christmas Eve, and will remain until January 2nd, and when the patients are well enough really to enjoy " a toon," as they call it, the nurses, students, and friends from outside will be only too pleased to play and sing and do all in their power to give them pleasure. The Christmas-tree entertainment will be held in the Board Room, as usual, on Friday, December 28th. Two dramatic entertainments, with which the festivities will close, will take place probably on Tuesday and Wednesday, January 1st and 2nd, in the new out-patient department.

### CHRISTMAS AT THE WOMEN'S HOSPITAL, CHELSEA.

Things are done in great style at Chelsea Women's Hospital, for the Ladies' Committee see that everything is of the best. "The turkeys," said Miss Heather Bigg, " are not common turkeys, but 'white turkeys,' which are rather rare, I believe, and the cakes are from Buzzard's, with chocolate icing." On Christmas Day the patients have a dinner and tea, with a tree and a present for everyone. Two friends may have tea with each patient, and there are crackers, candied fruit, nuts, oranges, grapes, and apples, and the nurses sing carols on each floor. At seven the nurses have late dinner—the only day in the year when this is possible. It takes place in the Board Room, and the Ladies' Committee usually present the matron with a magnificent basket of flowers to grace the table, while Dr. William Duncan, the senior physician, gives about half a dozen bottles of champagne. Each nurse has a present of a book. In order that the table may look as much as possible like one at a private dinner party Mrs. Heather Bigg lends some old silver candlesticks and other ornaments. On or about December 29th, the nurses give a concert to the patients, when they invite their friends to help with the music and songs. Miss Florence Christie has for two years coached the nurses beforehand, and she comes herself and brings one or two professional ladies and gentlemen to assist to make the concert a success. On January 5th there is still more entertainment: usually a conjurer is in attendance, and the patients' tea is carried round by the ladies and gentlemen who come to sing and play. This takes place from four to seven.

### CHRISTMAS AT THE VICTORIA CHILDREN'S HOSPITAL.

The little patients at the Victoria Hospital, Tite Street, Chelsea, are fortunate in having a kind friend who has for some years past given them a Christmas tree, with a present for everyone; and this year she has again promised them the tree. As the hospital also provide each child with a " stocking," they get two sets of gifts. There is another tree later in the week for the rest of the household. Extra plants and flowers are placed in the wards, but there are no evergreens on the walls. The out-patients have a tea, a present, and a Punch and Judy show.

### THE NURSES OF THE HOMŒOPATHIC HOSPITAL.

Five of the sisters and nurses will be absent from the Homœopathic Hospital at Christmas this year, for they are serving as reserve nurses in South Africa, and cannot return in time for the festivities. One is at Pretoria, two are at Mooi River, one at Bloemfontein, and one at Wynberg. It is expected that they may meet in Pretoria, as more nurses are being sent up on account of the fever. Each of the nurses who volunteered for the front received £1 for every year spent in the hospital, besides having her place kept open for her. One of the nurses had been there ten years. "They write every week," said Miss Brew, showing our representative a pile of letters on foreign paper, " and one of them says this morning how she wishes they could be with us for Christmas." The nurses look upon their hospital as a second home, and are always glad to return to it. " Even when they are married, they still come, and bring their babies ! "

### PROGRESS AT BOLTON.

The corner-stone of the building which is to accommodate the nurses of the Bolton Union Hospital at Fishpool has just been laid. The site of the new home is contiguous to the two blocks of infirmaries which were opened some time ago, and we observe with satisfaction the emphasis laid by speakers at the ceremony on the need of considering, not only the health and welfare of the patients, but those of the nurses also. For example, Canon Hoskyns, who has been Rector of Stepney, and knows the accommodation provided at the London Hospital for the nurses, said that " to have a well-conducted hospital it was absolutely essential that the nurses should be provided with properly equipped accommodation, and have surroundings which would enable them to lead cheerful lives, and partake of that corporate social enjoyment which they so much required." The home is to provide for a staff of 40 nurses, and, though plain in design, will not be lacking in anything requisite for the comfort or convenience of the inmates.

### BELFAST NURSES' HOME AND TRAINING SCHOOL.

In accordance with the requirements of the Local Government Board of Ireland, which one speaker at the annual meeting of the Belfast Nurses' Home and Training School said had been forced upon them, there has, during the present year, been an extension of the term of hospital training for the nurses of that institution from one and a half to two years. It is already admitted that the alteration is likely to be attended with beneficial results. Of course it involved the Society in increased expenditure, but the authorities of the Royal Victoria Hospital came to the rescue with a grant, and the receipts from the private nursing have this year touched the highest point in the history of the home, the loss on the year's work being reduced from £600 to £200. When the new Royal Victoria Hospital is in existence, its nursing will be carried out in the hospital, but the Society will then have to meet the ever-increasing demand for private nurses in Belfast. If they go on as they have been doing lately, they will soon be entirely out of debt, and there is every reason to suppose that the work will continue to obtain a substantial and increasing amount of support from the public.

### AN INTERESTING FUNCTION IN FIJI.

The Governor of Fiji, on behalf of the Queen, has invested Sister May Anderson, of the Colonial Hospital, Suva, with the Order of the Royal Red Cross, which, as our readers know, was lately bestowed by Her Majesty on Miss Anderson for services rendered to sick and wounded officers and men of the Royal Navy. The *Fiji Times*, in describing the function, says:—"The Governor having taken up his station in front of the assembly, Sister Anderson, in a prim-looking and immaculate uniform of light blue, relieved by a navy blue cape and the Geneva badge, passed into the ward with three other youthful nurses in her train, and was presented by the Chief Medical Officer to the Governor." His Excellency then delivered a brief congratulatory address, presented the Cross, and pinned it by its ribbon to the Sister's cape near the left shoulder. Miss Anderson, in some well-chosen remarks, and with considerable emotion, acknowledged the gratitude and appreciation she felt for the Queen's recognition of her work. She wished, however, to say that her gratitude and joy were necessarily tinctured by the reflection that it was through the sufferings of others that this honour had been bestowed upon herself. At the suggestions of the Chief Medical Officer, the Governor acceded to the request that the room in the hospital where the ceremony took place should be thenceforward called the "Admiralty Ward" of the Colonial Hospital, and declared it so named.

### A CRY FROM WHITBY.

In the interests of patients and nurses a protest comes to us from Whitby against the scheme for turning a very old house in a very crowded situation into a cottage hospital. It is proposed to spend £1,000 on the building; but if, as we are informed, the house stands almost in the river, there is always water in the cellars, and at low tide the smell from the mud banks in front is very bad, the description of "a beautiful situation" applied by a local paper to the site, will not render it suitable. At present the hospital is in Church Street, and although the accommodation is inadequate, a removal to Grape Lane will scarcely improve matters. It is very desirable in the interests of the District Nursing Association, which carries on operations in connection with the hospital, that the position of the permanent building should be unimpeachable in respect to healthiness.

### THE CASE OF A SUPERINTENDENT NURSE.

The East Preston Board of Guardians have unanimously agreed that Miss Rogers, the superintendent nurse, who has declined to resign, shall be dismissed in a month. It remains, however, for the Local Government Board to determine whether the dismissal shall hold good. They can, of course, insist upon her retention; but in the circumstances, with the whole Board of Guardians hostile to her, Miss Rogers could hardly be comfortable.

### GUARDIANS AND NURSES.

The Kettering Board of Guardians have voted a donation of fifteen guineas to the funds of the Kettering and District Nursing Association. All's well that ends well, and it is not a matter for regret that two members of the board should have raised objections to the increase of the donation, which has hitherto been twelve guineas. The contention of the dissentients was that the ratepayers did not approve the expenditure. Guardians are not to be blamed for taking a course they consider right to protect the pockets of the ratepayers. But in this case the overwhelming balance of evidence was in favour of the motion for the addition; and the assertion that the villages did not receive any benefit from the association was met by the statement that it relieves the Union of a certain expenditure, because it deals with so many of their cases. At the same time we share the regret that the surrounding villages are "suffering from want of nurses," and hope that a practical attempt will be made to supply their needs. It is quite true, as some of the Guardians said, that the Kettering Board cannot take the initiative.

### ROYAL NATIONAL PENSION FUN AND THE CHRISTMAS HOLIDAYS.

In order to save possible inconvenience to nurses coming up to town for Christmas, we are asked to state that the office of the Royal National Pension Fund for Nurses, 28 Finsbury Pavement, will be closed on Christmas Eve as well as on Christmas Day and Boxing Day. It is advisable that nurses should avoid posting their premiums on the 22nd. They should post them either before that day or on the 26th or 27th inst., so that they may be in the hands of the Secretary before the last day of the year, which is naturally, an extremely busy one.

### EXTRAORDINARY ACTION BY THE CROYDON BOARD OF GUARDIANS.

In another column will be found a report of the extraordinary course pursued by the Croydon Board of Guardians at their meeting on Tuesday. At the desire of several of the members, our representative says, the chairman allowed an anonymous letter from a nurse to be read in which, as it will be seen from our report, she discusses the question at issue between a majority of the guardians and the matron, with the obvious idea of confirming the Board in the attitude they have chosen to adopt. We, of course, agree with Mr. Owen that the letter ought not to have been read, and we commend the incident to the attention of the Local Government Board.

### SHORT ITEMS.

The winter entertainments for the in-patients at the National Hospital for the Paralysed and Epileptic commenced on Thursday evening last week, when an excellent programme was rendered. The next will be on January 17th.—The Committee of "The General Lying in Hospital, Lambeth," have recently expressed their gratification at another instance of the affectionate interest taken in the hospital by the past and present nurses of that institution. At the suggestion and through the exertions of Nurse Price, a fund of over forty pounds has been raised and presented to Miss Atkinson, the matron, to be employed at her discretion for the benefit of the hospital.—The Nursing Staff on the transport *Assaye* was composed of Sisters Jones, Chatfield, Halls, Wainwright (Mrs. Wainwright was formerly Sister at St. Thomas's Hospital and Dr. Wainwright was one of the medical officers on the *Assaye*), Sisters Jerram, and Paterson. Sister Draper was invalided home.—The s.s. *Yorkshire* has also arrived, with five Nursing Sisters on board: Sisters Wilson, Francis, Carruthers, White, and Theophilus. The first four are from the Infirmary, Cardiff.

### Lectures on Medicine to Nurses.

By H. A. LATIMER, M.D. (Dunelm), M.R.C.S. Eng., L.S.A.Lond.; Consulting Surgeon, Swansea Hospital; Past President of the South Wales and Monmouthshire Branch of Brit. Med. Assoc.; Past President of the Swansea Medical Society; Hon. Life Member of the St. John Ambulance Association, &c.

### SYMPTOMS IN FEBRICULA AND OF FEVER IN GENERAL.

THE symptoms of febricula being those common to the feverish state in general, it will be well if I devote a short time to detailing them and to rendering some explanation of their cause and significance. Concerning that great main feature of all fevers, rise of body heat, I need say no more, for I have already somewhat fully dealt with this phenomenon; but of some other symptoms of the pyrexial, or fevered state, I have as yet said little or nothing. Here, then, are tabulated symptoms, some one or more of which you will always meet with when fever is present.

Headache is generally to be met with, the severity of it varying from a sense of dulness, with slight pain, to a furious degree of agony. Different parts of the head may be the seat of the pain; and there are peculiar headaches in certain diseases which help the physician to diagnose the variety of illness which he is dealing with. But the great thing to be borne in mind with respect to simple continued fever is this: That with some people slight causes are sufficient to produce severe conditions of feverishness, whilst with others, such is the strength of their constitutions, and so great is the soundness of their nervous systems, that but little general disturbance follows upon irritations. The more one has an opportunity of studying temperaments the more one is struck with the fact that individuals have peculiarities of constitution which stamp an individuality upon their illnesses. Thus, one person will have a special proclivity to "catching cold," and a catarrh will result from an atmospheric change of so slight a degree that others will not have noticed that any change of temperature has taken place; and another person, whenever he becomes feverish from any cause, will suffer from a high degree of pyrexia. I suppose these sort of facts are at the back of the saying that "every man is a fool or his own physician at forty," it being assumed that, if he is ordinarily observant, by that age he will have learned what kinds of illnesses he is liable to, and what diet and regimen suits him when he is out of health.

All that I have been saying applies to the headache and other constitutional disturbances arising out of an attack of simple fever, for the same degree of irritation, upsetting half-a-dozen people, will result in half-a-dozen varieties of response to the same—responses not only in the matter of degree and severity, but in the region of the body selected for suffering. An easy way of reviewing symptoms is by grouping them according to the anatomical and physiological system to which they belong. If the physician associates functional acts of the nervous, the respiratory, the digestive, the vascular, the eliminative, and the muscular systems, and carries a mental picture of the same about with him, he can hardly fail to elicit from the patient, by objective and subjective examination, a true statement of what his sufferings are. Then it is for him to draw his own conclusions as to diagnosis, prognosis, and treatment.

Besides the headache from which sufferers from febricula are affected, the other symptoms of perturbation of the nervous system which may show themselves are sleeplessness, delirium, and shiverings. The shiverings I speak of may vary from slight sensations of chill to sensations of profound cold, and, in the case of children, even to convulsions. It is a point worthy of notice that the nervous constitution of a child is in so unstaple a state of equilibrium that an irritation which, in an adult will only cause shivering, may, in the earlier years of life, set up the graver complication of fits. Thus it may well happen—and often does happen—that in quickly-passing fever attacks during childhood, such seemingly slight causes as attacks of indigestion, and the irritation set up by teething, or the presence of worms in the stomach and intestines, will result in a general convulsive attack. Similarly, the disturbance of the circulation of blood in the brain may lead to excited nerve cell activity there which will reveal itself by perturbed thoughts and by rambling talk, passing on even into delirium. This, any of you who have attended sick children, are doubtless familiar with. In their sleep, when suffering from feverishness, they moan and toss about in their beds; and from time to time they suddenly call out names and short sentences, and if the excited brain action extends beyond those parts which act in thought and in speech to the centres presiding over muscular movements, these, in their turn, send down such strong currents of nerve force to the voluntary muscles that they contract in the spasmodic manner which constitutes convulsions.

As far as the breathing organs are concerned in the feverish state, unless they are especially affected by disease, the number of breaths drawn per minute keeps pace with the pulse beats, according to an ascertained rate of frequency. Under ordinary circumstances this correspondence is at about four or four-and-a-half beats of the pulse to every act of breathing, so that if the chest rises and falls eighteen times a minute, you would expect the pulse to beat from seventy-two to eighty times in the same space of time. This ascertained relative frequence of heart's action, and of breathing is known as the "pulse-respiration ratio," and if any marked derangement of it occurs it directs attention to one or other of the organs which are at fault, and which has shown itself to be so by its excited action. I need not say that rapid breathing shows itself quickly enough to anyone who will take the trouble to watch a few acts of respiration; still, for scientific accuracy, for recording the progress of an illness, and for observing whether treatment is succeeding or not, it is desirable to mark the pulse-respiration ratio carefully. It is a curious fact that, in the majority of cases, if people see that you are counting the pulse and the acts of breathing, the rapidity of these acts are increased, so it is well not to do this hurriedly but to wait awhile and to do it in the middle of an interview when the excitement of the doctor's arrival has passed away. Also, if a nurse is performing this duty, she should choose a time for doing it when the patient is at repose and not when the pulse and breathing acts have been accelerated by his having been moved about in bed. The respiration is counted either by watching the rise and fall of the bedclothes, or by placing the hand at the pit of the stomach, or just under the collar-bone. Then, with watch in hand, an observation can be made over a given period of time.

I have only now to deal with the disorders of the digestive and eliminative organs which occur in the feverish state, and these, important as they are, can be dismissed with no lengthy attention. Thirst is a prominent symptom in all fevers; it is due to several causes, such as increased body heat using up fluid in the system; to the locking up of secretions which also takes place, and to the more rapid acts of breathing which obtain, causing the sufferer to open the mouth and so dry the surface of the tongue and throat. The appetite for food and the power of digestion of the same also are affected, and because of the lessened activity of the secreting organs and of the torpor of the body generally, the urine becomes scanty in quantity and the bowels are constipated. Here, then, I have given you a review of the general features of the fever state; it will remain for me to describe the especial features of each variety of fever as I review such cases separately.

(*To be continued.*)

### Nursing at Wynberg Base Hospital.

INTERVIEW WITH SISTERS JONES AND CHATFIELD.

BY A CORRESPONDENT.

AN account of the nursing on board the transport *Assaye* has already appeared in the NURSING MIRROR, and so when I called on Sisters Jones and Chatfield we did not talk much about that part of their work. Both had, however, been for some months at No. 1 Base Hospital, Wynberg, and they had much that was interesting to tell. Sister Jones is an English nurse with an English training, while Sister Chatfield is now on her first visit to this country. She was born in Bloemfontein and was trained at Kimberley Hospital.

"But I am not a Boer," she explained; "my parents are English; people seem to take a long time to understand that I am simply a Colonial."

IN KIMBERLEY.

"I was within three months of finishing my three years," she went on, "and I am quite willing to go back and finish when I return to the Cape. When the wounded began to come in from Paardeberg we had a fearful time; they arrived in such numbers, and with many it was too late to try and save them. One who was shot in five places died a few minutes after he was brought in. I had just given him a few drops of brandy as he lay in the corridor. When the siege was raised I took charge of the Masonic Temple, and with a friend managed to get it into beautiful order, though at first we were in despair, for there was simply no provision at all for nursing."

"Did Kimberley people supply beds?" I asked.

"The De Beers Mining Company sent most of the things they were extremely kind. But it was terrible to see the men packed like sardines; when you wanted to sponge one of the patients you had to pull him out into the middle of the floor. It was hard not to be able to show how nervous you felt, too, when the shelling began."

Like the C.I.V., the Nursing Sisters in South Africa have had a good deal of practice in self-control; but it is not all who have had such special cause for anxiety as Miss Chatfield, for not only were her nearest relatives in momentary peril—in Kimberley itself—but her brother was taken prisoner by the Boers, and was within half an hour of being shot when a friend arrived and advised his life being put to the vote: he escaped by one vote.

AT THE BASE HOSPITAL.

After leaving Kimberley, Miss Chatfield was put in charge of the acute dysentery ward at Wynberg, where, by careful and conscientious nursing, she managed to pull many patients through. It was not, however, Miss Chatfield who told me this, but Miss Jones. "I can't tell you," she said, "how hard Sister Chatfield worked; I am sure many a man in Wynberg Hospital owes his life to her. We had terrible cases there, and no one knows the suffering the men went through, for many never 'went sick' at all until they were on the point of a relapse; they do so hate having to go into hospital. I think our work was harder than that nearer the actual seat of war."

SOLID WORK AT THE BASE.

"Less excitement and less change?"

"Yes; but just as good work was done. I had had a good deal of experience of nursing enteric here in England as a private nurse, before I went out last April, and it was this that made me volunteer. I wanted to get more practice in enteric, and I also wanted plenty of hard work."

"You did not want to go to the front?"

"I should have liked it, but there were so many who wanted it more than I did."

"Have you been at Wynberg throughout?"

"Yes; I was put in charge of the acute enteric ward at No. 1 under Sister-superintendent Garrick. She is quite one of the best and wisest matrons I have worked under—an admirable woman in every way. She is averse to changing the sisters in the wards, and consequently I got to know my patients thoroughly—a very necessary thing in fever."

"Some civilian nurses seem to have found the Army system difficult to work under; you do not share that view?"

"No, although I am a civilian nurse myself. I made many mistakes at first in matters of detail, and no doubt some of the lower rank of officials were annoyed, but the P. M. O. and superior officers always understood, and were most kind and easy to get on with."

LIVING SKELETONS.

"It is extraordinary," continued Miss Jones, "what recoveries were made at Wynberg, At first it was terrible—men dying all round. This was in the spring, when illness was at its worst. But later I saw men come in like skeletons, sent down from Bloemfontein and other centres. One particularly impressed me. His was a case of dysentery, and he had been ill for months; he looked so old—any age between seventy and a hundred—with tightly-drawn skin and horrible bed sores. Well, when that man was discharged he looked perfectly blooming with health, and young, too; he was only twenty-one!"

COMING HOME.

The voyage home was uneventful; all the patients benefited greatly, and not one was lost at sea. The unpleasant features of the time may be summed up in one sentence: "The *Assaye* doesn't roll," said Sister Jones, "but she pitches." Being an Indian boat she had punkahs—an enormous boon in the tropics. The point specially noticed by the nurses was the quick and workmanlike way in which the cot cases were disembarked at Southampton, and they spoke, as others have done, of the extreme kindness of the disembarking staff under Colonel Charlesworth.

Both sisters spoke most highly of the R.A.M.C. in South Africa; they think that in cases where nurses have found it difficult to work under the army system it has been for want of tactfulness and willingness to adapt oneself to new conditions. "If your orderly likes you," they said, "he will do anything you tell him." They are very much fatigued after the trying times they have gone through, and Sister Chatfield, who acknowledges that she has worked hard, told me she did not know until she arrived in London how tired she was. She is, however, having not only a rest, but a thorough change; and she is seeing theatres as well as hospitals.

### To Nurses.

WE invite contributions from any of our readers, and shall be glad to pay for "Notes on News from the Nursing World," or for articles describing nursing experiences, or dealing with any nursing question from an original point of view. The minimum payment for contributions is 5s., but we welcome interesting contributions of a column, or a page, in length. It may be added that notices of entertainments, presentations, and deaths are not paid for, but of course, we are always glad to receive them. All rejected manuscripts are returned in due course, and all payments for manuscripts used are made as early as possible at the beginning of each quarter.

### Christmas Day, 1900.

I WRIT about last Christmas time
An' told you all the fun—
Wot we had in the 'orspital,
An' all the things we done.

This year we got a bigger tree,
Our ward's the best of all:
There's picturs of our generals
An' flags upon the wall.

" The Soldiers of the Queen " we sing,
Wot's fightin' far away;
You bet they'll think on us at home
A' keepin' Christmas Day.

We're singing that, 'cos of the war,
Last night all on us tried:
The nurses, they sang " Saints of God "
For them wot fought and died.

Them babies make a awful row,
They shout with all their might;
*They* don't know nuffin' 'bout no war,
*Their* dads ain't bin to fight.

*My* dad's a soldier of the Queen,
Like them wot's in the song:
And he's a corporal—he is—
Oh, ain't he big an' strong!

An' when they dress my back I try
To be right brave like him;
'Cos Sister holds my hand and says,
" Remember Daddy, Jim ! "

I haven't cried not once this week,
But when them tubes go in
I have to think of soldiers hard,
Or else I might begin.

It wasn't Bores what hurt my dad,
But " 'Terics " what they said;
An' he's been in a 'orspital,
An' very nearly dead.

This afternoon won't be no fun,
My dad won't see the tree;
Them kids has all got visitors,
But none ain't come for me.

My mother she ain't here, an' now
My back's a hurtin' bad;
I'm tired of trying to be brave,
He'll never come, won't Dad.

She said, did sister, " Jimmy, think
So many wait in vain
For those who never can come home
For Christmas time again."

Who's that a comin' down the ward?
It's Mammy, walking fast,
An' someone with her. Hip hooray!
'Tis Daddy, home at last.

An' did the 'Terics let you go?
An' are you wounded bad?
Oh let me feel your khaki coat.
Look! Sister, here's my dad.

And he's come from the war, has Dad,
His ship got in last night;
He's just in time. Look, Daddy, look,
The tree is all alight.

It's shining bright with twinkling stars
All sparkling in the green;
They're cutting off the presents now,
The best there's ever been.

Oh lift me up an' carry me,
My back don't hurt no more.
There never was no Christmas Day
So good as this before.

### Appointments.

TROWBRIDGE COTTAGE HOSPITAL.—Miss Caich has been appointed nurse-matron. She was trained at the London Hospital.

WREXHAM INFIRMARY.—Miss Mary Barwick has been appointed matron. She was trained at Leeds General Infirmary, and has since been matron of Bridgend Cottage Hospital and Pembrokeshire Infirmary, Haverfordwest.

URMSTON COTTAGE HOSPITAL.—Miss Lucy A. Cornwell has been appointed matron. She was trained at the General Hospital, Nottingham, and was for five years sister-in-charge of the male medical cases at the same hospital. Since then she has been matron of the Sidmouth Cottage Hospital for two years, and district nurse at Abingdon for three years.

VICTORIA HOSPITAL, WORKSOP.—Miss E. Langstaffe has been appointed matron. She was trained at the London Hospital. She has since been charge nurse at the Devon and Exeter Hospital, district nurse at Dulverton, nurse at Bridgwater Infirmary, sister at East Ham Borough Sanatorium, and district nurse at Littleport and Luton.

WALSALL AND DISTRICT HOSPITAL.—Miss G. H. Sked has been appointed matron. She was trained at the Royal Infirmary, Edinburgh, and the Royal Infirmary, Liverpool. She has since been assistant matron at the Royal Albert Edward Infirmary, Wigan, and matron at the Royal Isle of Wight County Hospital, Ryde.

### Presentations.

CITY HOSPITAL, EDINBURGH.—On the evening of Saturday last the medical nursing staff of the Edinburgh City Hospital assembled in the recreation room in order to present a wedding gift to Nurse Florence Mather on the occasion of her marriage. The medical superintendent doctor spoke most warmly of Nurse Mather's work during her four years in the hospital, mentioning that twice she had acted most successfully as matron of the smal-pox hospital. He said that the gift, which consisted of a handsome teapot, cream jug and sugar basin, sugar tongs and toast-rack, was a spontaneous gift of affection and regard from the whole staff. At Dr. Ker's request Miss Sandford, lady superintendent, made the presentation, cordially endorsing Dr. Ker's remarks, and testifying her own gratitude for the good example Nurse Mather had shown by her loyal and faithful work. Miss Sandford, while wishing Miss Mather every happiness, said she would be much missed by the whole staff. The evening was concluded by a very pleasant concert among the nurses.

### Our Christmas Supplements.

WITH the present issue of the HOSPITAL NURSING MIRROR are presented five handsome plates: "Within the Wards," " Christmas Tree Corner," "A Ward in the Roof," "Delicate Tracery," and " A Corridor." Also a supplement containing a series of sketches of Christmas in the home and Christmas in the hospital.

### Christmas Books.

Christmas usually affords leisure for reading, and no gift is more popular than a suitable book. The difficulty lies in selection, and the main difficulty is to find the time to make a search amongst the numbers from which we have to choose. We have endeavoured to help our readers by classifying the books which have come under our notice, so that the book suitable to the standing of the reader may be more readily discerned.

### BOOKS FOR LITTLE CHILDREN.

The Tale of the Little Twin Dragons. (Macmillan & Co.)

The Little Twin Dragons went out in search of little Prince Valentine who was lost. Their exciting adventures are told by Miss S. Rosamond Praeger in a highly entertaining and clever manner through the medium of pen and brush. The twins always wore distinguishing ribbands round their necks, until after a deadly fight with a knight whom they worsted: then one of them bound its head up in a piece of the knight's coat, which rendered her appearance henceforth more attractive than ever.

The April Baby's Book of Tunes. (Macmillan and Co. Limited.)

Most of us are familiar with that fascinating book "Elizabeth and her German Garden," and so we open the little volume by the same authoress with an expectation of pleasure which is fulfilled as we peruse it. Round the three babies, April, May, and June, who are introduced to us, is woven a pretty rigmarole, in which the time-honoured nursery rhymes are introduced, with and without their tunes, and rendered more graphic by pretty coloured illustrations. The little book will be a favourite in the nursery. Strange to say, the "tunes" form the smallest portion of the volume.

A Noah's Ark Geography. (Macmillan and Co., Limited.)

The Noah's Ark Geography is delightful, especially the pictures in colours, which are cleverly drawn by Miss Mabel Deamer, who is also the authoress. The print is fine and large, and the language clear also, and little people will find themselves taken by pleasant stages for a visit to all parts of the world.

Fiddlesticks. (C. Arthur Pearson, Limited.)

Fiddlesticks, by Hilda Cowham, is quite one of the Christmas books this year. It is just what the young ones like—nearly all pictures—and what letterpress there is is very large, and all comes out of the immortal nursery rhymes. It is a work for the nursery and the drawing-room.

The Other One. (C. Arthur Pearson, Limited.)

This is a delightful little story of the adventures of three kittens told by "The Other One," so named because his more attractive brother and sister inspired the only names which occurred to the imaginative little owner of the kittens.

The House that Grew. (Macmillan and Co., Limited.)

Mrs. Molesworth tells us a pleasant tale about some nice children who made a happy home for themselves in a temporary iron building, when their parents had to part with their family mansion through loss of money. The house "grew" because several friends and relations who joined them in their humble dwelling brought their own huts, like snails, with them.

### BOOKS FOR GIRLS.

The Girls' Little Book. (Messrs. Skeffington and Son.)

This admirable little book for girls, by Miss Yonge, has come out in a new and very suitable dress this Christmas. Even if the choice of literature for our girls has already been made, this small book should be added to the Christmas gifts. It is full of wisdom attractively imparted; it is a form of literature most needed but much overlooked in the present day.

In a Cathedral City. (Chatto and Windus.)

This is a new edition of Miss Bertha Thomas's most pleasing story, "In a Cathedral City." The tale is told of a brave little woman who finds a refuge in the Cathedral City, where she earns her bread as a dressmaker. All the characters of the tale are interesting and the tone of the book wholesome.

A Measuring Eye. (S. Partridge & Co.)

A simple story, simply told in a fresh and interesting manner. The struggling little authoress who lives at Beech Cottage, and is robbed by her worthless brother—commands our sympathy, and we rejoice that she found so good a friend in old Jonah, her landlord. In the end the heroine Ruth Marten meets with her deserts, and eventually her brother dies repentant. Miss Elizabeth Stuart-Langford has placed before us a very pleasant little book.

Rhoda. (Thomas Nelson, Limited.)

This is a tale of five girls who found themselves possessed of the smallest of incomes on the death of their father, who had brought them up to every luxury. These young people are very proud, and resolve to disappear from the horizon of their friends to avoid any patronage or pity. They make a home in London, and then the struggle begins, more especially for Rhoda, the self-sacrificing elder sister, who alone possesses an ordinary amount of common sense. All's well that ends well, and the sisters are eventually discovered by their friends and restored to happiness and comfort. We are indebted to E. S. Haverfield for an interesting though simple chronicle of the adventures of the Misses Vinning.

Daddy's Girl. (George Newnes, Limited.)

"Daddy's Girl" is almost too delightful a young personage to be true. She is but eight years old, yet she has the most clear and direct judgment on all the affairs which centre round her little life, which, alas! comes accidentally to a pitifully abrupt termination. Mrs. Meade manages to lessen our regret by portraying the good resulting to both parents of the little girl through the lessons of her death-bed. The little narrative is charmingly told, and we put it down with a feeling of affection for "Daddy's Girl."

Notable Work by Notable Women. (S. W. Partridge and Co.)

In this age, when youthful self-satisfaction is so very prominent, a little book like this is a valuable addition to the literature for young people. No lessons are so valuable as living demonstrations, and the lives of Mrs. Gladstone, Mrs. Fawcett, the Baroness Burdett-Coutts, Lady Henry Somerset, and Miss Sarah Robinson show results which call forth admiration and stir to emulation. All these are narrated in a neat little volume by Miss Jennie Chappell which should find its way into the hands of "Our Girls" at Christmas.

### BOOKS FOR BOYS.

China of To-day: The Yellow Peril. (Messrs. George Newnes, Limited.)

This is a thoroughly up-to-date album, illustrating the principal places, incidents, and persons connected with the crisis in China. It commences with a view within the British Legation, the scene upon which the interest of all England was focussed for so long. This picture alone is enough to ensure a welcome for this interesting volume. The clearly defined plans showing the situation of buildings the names of which we are familiar with in Pekin are more instructive than the best of verbal descriptions. The whole contents of the album are attractive and instructive, and it is bound in an artistic and striking manner.

THE RED, WHITE, AND BLUE. (Thomas Nelson and Sons, London and Edinburgh. Price 2s. 6d.)

THIS is the tale of all kinds of craft afloat under the Union Jack. It contains beautifully coloured illustrations of all the principal types of ships, and a frontispiece also in colours of the Naval Review of 1897. The letterpress is in large clear type and simple language that all young Britons can understand. Of course a large space is devoted to our navy; but all kinds of shipping is illustrated and described, as well as the main features of the different services. The fine representation of *La Marguerite* on her way to Margate, and of the *Powerful* in Durban Bay, and Nelson's *Victory* will all three be recognised by many young readers with interest. The fine and handsomely got-up volume will form a remarkably inexpensive gift, which will be welcomed by every small boy.

AFTER WORCESTER. (D. Nelson and Sons.)

THAT romantic period which followed the battle of Worcester has been selected by Mr. Everett Green in which to tell his story of the royal fugitive Charles II. He has made the best of his material and woven a fascinating romance, full of all the stirring incidents which marked those eventful times. The book will instruct young readers, whilst giving them a recreation of an absorbing character.

ROY: A TALE IN THE DAYS OF SIR JOHN MOORE. (C. Arthur Pearson.)

ROY is a very charming tale which depicts more especially the life of English prisoners detained in France when England was at war with Napoleon. Miss Giberne, the authoress, has taken pains to draw the environments and conditions from actual records, so that the pictures she gives are not only interesting, but they are true. She is full of enthusiasm and admiration for that splendid soldier Sir John Moore, who was lost to his country in the midst of his career, and she inspires her readers with renewed recognition of his virtues. For an appreciation of Napoleon one must go elsewhere.

### BOOKS FOR EVERYONE.

A SISTER OF THE RED CROSS. (Thomas Nelson and Sons.)

THE heroine of this story is a nurse, and the main scenes take place in Ladysmith. The authoress is Mrs. L. Meade, and these facts will recommend the book to many readers, especially those belonging to the general public. To these latter the stirring incidents of the story will appeal undisturbed by the technical incongruities which must mar it in the eyes of professional readers. Sister Molly, secures the Red Cross, and Gavon Keith, on whom is bestowed the V.C. after many trials and threatened separations, marry happily in the end.

A SUGAR PRINCESS. (Chatto and Windus.)

IN this essentially sordid age it is quite refreshing to meet with the chronicles of three such unspoilt beings as the "Sugar King," his son and daughter, and Carl Meyer, the hero of Mr. Albert Rosse's story. The episodes of the story arise out of the suspicions which are put into the mind of Carl Meyer's patron by his old friend. He persuades him Carl is mercenary and unworthy, and advises him to put Carl to the test before making the young man his heir. Carl, however, is of sterling merit, and after many vicissitudes is restored to his adopted father's confidence, and wins the love of the "Sugar Princess."

A SON OF AUSTERITY. (Ward, Lock, and Co.)

A SON OF AUSTERITY is a powerful book, and the unusual subjects which the author has chosen to weave into his tale have a fascination from the way in which they are treated. The hero, Paul, was born in the workhouse. He is the "Son of Austerity." His mother was deserted by his father, and her whole life embittered. Later the father returns, and the son has to choose whether to cast in his lot with father or mother. A tragic vein runs throughout the story. The cloud falls on the secondary figures also. The little blind and misshapen sprite who listens to the songs of Allan Carry, the dwarf gardener, and calls him her Fairy Prince, appeals to our imagination and sympathy. There is much sadness throughout the tale, but in the end there is justification and happiness.

### The Crisis at Croydon Infirmary.

BY OUR OWN CORRESPONDENT.

THE Croydon Guardians sat on Tuesday, and it was intimated that no communication had yet been received from the Local Government Board. In the morning a special meeting of the Infirmary Committee was held to further consider the question of appointing a superintendent of nurses who, if the Local Government Board permits it, is to control the nurses in the place of the matron. This Committee, however, presented no report. During the sitting of the Board the Chairman said he had received, as Chairman of the Board, a letter from a nurse on the Infirmary difficulty, and it would be for the Board to say if this letter should be read.

Several members desired that it should be, and the Clerk read as follows (withholding the names):—

TO THE CHAIRMAN, CROYDON BOARD OF GUARDIANS.

Bedford House, Hastings, December 5th, 1900.

SIR,—I, as a late nurse, have taken deep interest in the crisis at Croydon Infirmary, and would venture to give my opinion and experience.

A matron's signature should *not* be considered at all necessary for a nurse's certificate. Also the sooner the supreme powers of some matrons are subjected to the higher authorities the better.

I have worked under some matrons, two of whom could almost be compared to angels, whereas others seem to enjoy making the lives of their subordinates miserable. I had worked under the one for a period of two years, during which time she had never had occasion to report me to the medical superintendent, neither to the Committee, for being late, or any other crime nurses are prone to; and yet when I left (resigned of my own free will), not called upon to do so—my certificate signed by the medical superintendent, also the chairman—out of sheer vindictiveness, the matron had the impudence to put "General conduct fair."

Had I allowed it my people would have made a commotion at the time. But I put it down to her nasty disposition. I knew I was not a favourite of hers. I could never toady to her as some did. I destroyed the parchment. I knew I should succeed better without than with such a piece of injustice. Can anyone imagine anything so objectionable as nurses being tyrannised over to such an extent?

The writer then gave particulars of her service, including mental, fever, and maternity nursing and proceeded:—

One old lady, out of gratitude for my untiring unselfish love of my work provided for me for the rest of my life. But even now I am too fond of nursing to give it up. And yet a woman once felt too conscientious to give me the certificate I earned. I know nothing of the three nurses, whom your matron refused to sign the certificates. But if they were so uncultured, selfish, and insolent, why were they allowed to remain their time—(several guardians interposing: Hear, hear)—or why were they not reported? Oh something surely requires investigation here!

I apologise for troubling you, but the words of Him who said "Do unto others as ye would they should do unto you" are ever before me.

To try to right a wrong is indeed a pleasure to

Yours faithfully,

Sister * * * *

Mr. W. H. J. OWEN said the letter had better not have been read. It was most unfair that such a letter should have been read.

Mrs. WILLIAMS inquired if the writer had ever been one of the Croydon nurses, but the Clerk replied that he did not know her.

The letter was referred to the Infirmary Committee.

### Christmas Novelties.

By Our Shopping Correspondent.

### THE BEST HOSIERY.

A visit to the establishment of Mr. Fred Penberthy, 390, 392 Oxford Street, at this period of the year cannot fail to be, in every sense of the word, satisfactory. Very critical and very hard to please must be the customer who feels that she wouldn't, if her purse allowed it, buy twice over the many charming articles that meet the eye. Needless to say everything is of the very best quality, and remarkable alike for delicacy of design and excellence of workmanship. Gloves and handkerchiefs especially riveted our attention. In the former the assortment is enormous, and at prices to suit every purse. As a Christmas present, perhaps, nothing could be found more acceptable than a pair of reindeer-skin gloves lined with soft fur or rabbit's down, which for softness of texture and warmth are simply unrivalled. The price of these delightful novelties is only 8s. 6d., and they wear for ever. Embroidered and lace-edged handkerchiefs next claim the attention, and how dainty and coquettish they are; 2s. 11d. will purchase one worked in a garland design with fine lace edging, and there are others trimmed with Duchesse, Brussels Point and Maltese real lace, hand embroidered, at prices varying from 18s. 6d. to 30s. Stockings are another speciality, the designs in which are perfectly bewitching. I quite lost my heart to an especially charming pair in fine black silk, embroidered up the front with little bunches of forget-me-nots, the price of which I learnt, with surprise, was only 8s. 11d. Turning to the more useful articles for which Mr. Penberthy is equally celebrated, I found a charming wrapper in nun's veiling, with a faint pink silk stripe running through, faced with soft pink washing silk. It was a fairy-like garment, but warm enough for its purpose. Another attraction in this line was a pale blue striped dressing jacket, with revers of pale blue silk. The large variety of night-dresses leaves nothing to be desired, those in Vyella flannel being of the softest and warmest description, and eminently suitable for invalids. The grey angora vests are worth inquiring after, also sleeping socks in the same luxurious material. In conclusion I need only refer to the combinations with cholera belt introduced into the body of the garment which ensures a more comfortably fitting article than wearing the same in three separate pieces.

### A CHRISTMAS BAZAAR.

At this favourite rendezvous the display is more than usually attractive this year, and those of our readers who find themselves in the neighbourhood of the Edgware Road will be well advised to call at Messrs. Garroulds' and inspect their numerous and fascinating novelties. In the Christmas bazaar will be found many a little souvenir suitable for hospital and institution use. Especially in the matter of ward decoration, where often the ingenuity as well as the purse of the sister is taxed, I was struck by the excellent assortment of flags and shields suitable for the purpose. This mode of decoration has many advantages. For one thing it will go on from year to year, and for another they are always handy for any special occasion that may arise during the year. Our successes in South Africa caused many of us to regret that we had not got stowed away some of these tokens of national rejoicing, as the difficulty was to procure them in sufficient quantities at a reasonable sum when the occasion arose for their use. Toys and presents also are there in luxurious abundance and at the most reasonable prices. Paper-weights of quaint design at 10½d., writing-cases from 1s. 11½d. upwards, music-cases in excellent imitation crocodile leather—all are evidence of the full understanding tha Messrs. Garroulds possess of the taste and purses of their numerous customers. I admired some really well-got-up brass photograph frames at 10¾d. each which would give great pleasure to many a poor patient. Fancy aprons and lingerie of all sorts are other successful lines, and are well worth inspecting on their own merits alone. In the outfitting department I noticed a charming little bonnet, pointed in front and trimmed with a becoming bow of black velvet. The name of this little gem is the "St. Ives," and is priced at 10s. 11d. This I consider to be one of the neatest nurses' bonnets in the market. Of nursing requisites there are any amount—pincushions in red morocco of various shapes, wallets, and bags are more attractive than ever—and last but not least I would draw the attention of our readers to the sweet little silver watch with red enamelled cross on back which would make simply an ideal Christmas present.

### COMFORTS FOR INVALIDS.

This well-known firm in catering for invalids is to be congratulated on the excellence and variety of the articles included in the catalogue. The "Harrogate" chair is quite a novelty, and one that is certain to become popular as its advantages become more known. It is made in very strong cane, and can be used either as a chair or as a lounge by simply drawing the foot end out from under the chair when required to serve the latter purpose. The back also is adjustable, and the cost ranges from 27s. 6d. to 33s. 6d., so it is by no means out of the way. There are several designs in the popular "Ilkley" couch, and the price varies with the quality. The "Pompadour" easy-chair is an excellent arrangement for a wheel chair, as it can be easily propelled in any direction by the occupant. It is provided with a shaped footboard covered with carpet, making it very comfortable, and the price is £6 18s. 6d. There are also some good useful carrying chairs in cane, which renders them very light and easy to manipulate. The "Merlin" chairs are likewise worthy of notice, as they are remarkably well finished off and will commend themselves from their well-known advantages over other chairs of the kind to the favourable consideration of the public.

### THE BEST EAU DE COLOGNE.

It has been my pleasure for many Christmases past to call attention to Mühlens 4711 Eau de Cologne as a welcome Christmas present. I have always found it the best Eau de Cologne, and I believe that this is now the universal opinion. All the perfumes and toilette specialities manufactured by the same firm are excellent in quality, and they can be purchased, if desired, at the depôt in New Bond Street.

### PLEASING VARIETIES.

Those who are in search of Christmas presents, handsome in design and reasonable in price, cannot do better than pay a visit to the establishment of Messrs. Oetzman and Co. in the Hampstead Road. For presentation purposes the variety is as large as it is attractive, and there are still several of the smaller sort to entrance the mind of the purchaser. In sterling silver the assortment is most seductive and the prices most reasonable. An elegantly-designed toast-rack can be bought fo[illegible]s. 6d., and a charming case of afternoon teaspoons in morocco case for for 21s. 6d. A five-pound note will buy a sweet little tea service, while a richly-chased bowl or sugar basin with tongs can be had for 63s. I admired very much a small Queen Anne cream ewer, and was surprised to hear it could be had for the comparatively trifling sum of 11s. 6d., while a "Dog Toby" jug, 2½ inches high, only costs 13s. 6d. There are a variety of ornamental dishes, cigarette cases, ash and pin trays, also candlesticks and vases. The toilet requisites are very attractive, and appear in all shapes and designs. There are also photograph frames and a number of other beautiful and useful articles.

### CHOCOLATE AND COCOA.

IT is difficult to say anything which has not already been said in praise of Messrs. Cadbury's excellent productions. They are tempting, wholesome, pure, and nutritious. English people who go further afield with the idea that in purchasing foreign chocolate and cocoa they are securing something very superior to the English manufacture of Messrs. Cadbury make a great mistake. Messrs. Cadbury produce both of the highest excellence. The very finest chocolate can be had of their manufacture at less cost than is asked for Continental products. Every year their sweetmeats improve in quality and variety.

### HOUSEHOLD THERMOMETERS.

THE very unattractive appearance of most thermometers is responsible, in some measure, for their neglected use in the household. Messrs. Taylor Brothers, of 103 Hatton Garden, E.C., have produced a thermometer which does away with the objection. Their radial scale thermometer is quite a pretty decoration, which is arranged for suspension from a chandelier or gas bracket. The tube and top end are protected by a decorative casement of nickel or oxidised silver, and the reading is rendered as distinct as possible by the tracing in white of the measurement on black wooden wings. Messrs. Taylor make all kinds of thermometers, of excellent workmanship, but it would be difficult to find a rival to their radial scale thermometer.

### The Nurses' Bookshelf.

URINE TESTING FOR NURSES. By J. BEARD, M.R.C.S.Eng. L.R.C.P.Lond. (Bradford: Milnes and Longbottom 1900. Price 6d.)

THIS little book aims at teaching nurses how to apply the common urinary tests so that they may be able to roughly examine for albumin, sugar, bile, and blood. First we find a description of a normal urine and a statement of some of the changes which it undergoes in disease in regard to quantity, colour, smell, specific gravity, &c., and then certain tests for albumin, sugar, bile, and blood are described. Considering how very slight an outline of the subject is given, we think more care might have been given to its production. The typographical setting out of the subject is lacking in system, and the wording is sometimes obscure. To tell a nurse to add "a little" acetic or nitric acid is not sufficiently definite. On page 6 we are told that the urine is diminished among other conditions "in a disease called aritiosis." Can it possibly be that this is a misprint for [illegible]rhosis? On the same page we find "urethea" for "urethra." Altogether this little book is a careless production, and one which is not likely to be of use to a properly-trained nurse.

### Help the Nurses to Help the Sick.

**Royal National Pension Fund for Nurses,** 28 Finsbury Pavement, E.C.—This Fund has during the past year maintained the uninterrupted success which has attended it ever since its establishment. The number of members who have joined the Pension Fund in 1900 shows a most satisfactory increase. Over £1,500 has been distributed in sick pay; a fact which speaks eloquently as to the blessing which this branch of the Fund's work must be to nurses, more especially, of course, to those working on their own account. About £4,300 was paid away in pensions and bonuses in 1900, and it is interesting to note that this amount, as compared with the previous year, shows an increase of not less than 43 per cent. The premium income—*i.e.*, payment by, or for, nurses—was over £70,000.

**The Junius S. Morgan Benevolent Fund** is an auxiliary to the Royal National Pension Fund for Nurses, was founded through generous contributions from nurses themselves, and raised to handsome proportions by the munificence of the Morgan family and many other friends to nurses. The work is done by volunteers, under the supervision of an influential committee, which devotes time and care to the investigation of claims and the relief of urgent cases of distress amongst the policy-holders in the Pension Fund. Hon. Secretary, Miss Rosalind Pritchard.

**East London Nursing Society.**—The object of this society is to nurse the sick poor in East London in their own homes by means of trained resident nurses, each nurse living in the parish in which she works. The extent of the society's useful work is shown by the fact that in 1899 the staff of 31 nurses attended to 5,306 persons, to whom 120,022 visits were made. Annual subscriptions and donations to the general fund are asked for. Secretary, Mr. Arthur W. Lacey, 43 Rutland Street, New Road, Commercial Road East, E.

**Queen Victoria's Jubilee Institute for Nurses.** Offices: St. Katharine's Precincts, Gloucester Gate, Regent's Park, N.W.—The Institute trains nurses in district nursing, and supplies nurses to affiliated associations for the sick poor in their own homes. Applications for information should be addressed to Miss Peter, the General Superintendent. Nursing associations in England, Scotland, Ireland, and Wales are affiliated with the Institute.

**Up-country Nursing Association For Europeans in India.**—The chief object of this association is the provision of skilled nursing for Europeans, especially civilians, in the up-country districts of India. The association in London selects the nurses, and all expenses are paid. Hon. Secretary, Major-General J. Bonus, R.E., Clapton Court, Crewkerne, Somerset.

**The Colonial Nursing Association,** the Imperial Institute, S.W.—This valuable association was founded four years ago to supply trained nurses in the Crown Colonies and small British communities in foreign countries. Since the foundation 80 nurses have been despatched to various parts of the world, grants in aid being made where it is clearly shown to be impossible for the residents unassisted to bear entire cost of passage moneys, salaries, and maintenance. It is one that appeals to the sympathies of all, for which family is there that has not some members in distant lands, building up the Empire, and fighting with the sickness that comes with rough faring and undrained country? The Hon. Secretary, Mrs. Debenham, will be glad to receive contributions, especially as an effort is being made just now to extend its benefits to the poorer colonies.

**"The Hospital" Convalescent Fund.**—The object of this fund is to provide rest for weary workers amidst suitable surroundings, without any of that anxiety about ways and means which retards convalescence. Since the establishment of it many tired and delicate workers have enjoyed a much-needed change of air such as they could not possibly have secured for themselves without help. Experience has proved that it is better to let the nurses have a choice of locality rather than to send them to one settled place, and nurses are accordingly sent to all parts of the country. Contributions which would increase the field of usefulness are invited by the Hon. Secretaries, care of the Editor of THE HOSPITAL.

### Nursing in an Indiana Hospital.

By a Correspondent.

There recently appeared in the Mirror a series of articles on nursing in America. I thought them very interesting, and with the idea that English nurses may like to know a fellow-worker's impressions of a hospital in Indiana I send an account of my experiences. I by no means wish to convey the impression that all American hospitals are the same; in fact, I expect they vary as much as ours do. The hospital in Indiana to which I allude is a large, red-brick building, standing in its own grounds, which are not well cared for like English hospital grounds. There are no flower-beds, nor anything pretty and bright about them; indeed, there is an absence of decoration of any sort in the whole building, which is the more striking to those familiar with our cheerful English hospitals.

### Bare Wards.

The wards are large, and well lighted by big windows and by electricity at night; but there are no flowers, no pictures, no plants, and no tastefully decorated tables. Simply the beds and a few chairs, some of which are cane lounging-chairs. The surgical ward to which I was attached is divided into two parts, called respectively the north and south wards. In the north ward the bedsteads are of green iron, surmounted by straw mattresses about 2 feet thick, making the total structure nearly breast-high. These beds are of course quite hard, and are used for fractures and diseases of the lower limbs. The beds in the south ward are of white-enamelled iron, with the well-known wire spring mattresses. A folded blanket is laid over the wires, then the mackintosh and sheet, which is turned in under the blanket and kept in place by safety-pins.

### No Diet-Sheets.

On all the beds the top clothes are pinned together. Of course the pins, which are all nickel-plated, are not visible; I never saw a brass safety-pin in the hospital. It naturally takes as long again to make the beds as it does at home. There are no bed-boards nor diet-sheets. The doctors write all directions for treatment in one book, and order all medicines in another. Those patients who are able go down to the dining-room for their meals; and for those who are unable the doctors have to write tickets for trays, to be sent either from the ordinary patients' kitchen or from the "diet" kitchen.

### The Nurses' Room.

Each ward has a "nurse's" room, and in some wards this room is quite separate, but in the ward I was in it forms a corner of the south ward railed off, and commands a view of both divisions. In it is a wash-stand for the use of nurses and staff, a refrigerator, a writing-table with drawers for the books, charts, &c., and a cupboard containing a small dispensary.

### Nurses Compound Medicines.

Unless a very complicated prescription is ordered, the nurses compound the medicines dose by dose. This seems a dangerous proceeding when one considers that often a nurse of only six or eight weeks' standing is left in charge with perhaps a dozen different medicines to give. I myself saw a nurse of six' weeks standing give a coloured girl a tannin gargle to *drink*. I mildly suggested that it was not meant to be taken, but my interference was resented. Of course, had it been a poison I should have insisted on the superintendent of nurses being fetched. As it was, I privately reported the occurrence both to the nurse in charge and to the medical superintendent. No temperatures are taken unless ordered by the doctor, and then they are every four hours. If a patient seems worse, it is not taken more frequently unless the doctor has been and ordered it to be done.

### Charts and Dressings.

Charts are only used in *very* serious illnesses, and then everything is recorded on them. These charts are kept in cases, hung on the walls in the nurses' room. No dressings are done in the wards. To each surgical ward a small surgery is attached, fitted up with all things necessary for dressings, minor operations, and obstetric examinations. Every corridor has two or more ambulances on rubber-tyre wheels, and the patient is wheeled from his or her bed to the surgery. For the male wards there is an orderly, who keeps order among the men and boys, and also assists in the lifting of heavy patients of either sex.

### The Operation Table.

All the floors are polished, and the coloured porters rub them up every other day. The corridors are done every day. The building is heated by steam radiators, which to English ideas are a very poor substitute for coal fires. Of course, the theatres and all pertaining to them are of the newest and most modern description. The metal operation-table in use in England is a copy of the American one, if it does not actually come from America. The table in this hospital has a hot-water tank which, when filled, keeps the patient warm, and is, to my mind, a very great improvement.

### Special Dresses for Nurses.

Whilst conducting operations not only do the surgeons wear white linen over-alls, but the nurses also have special dresses made of the same material, quite plain, but full from the neck, and fastened round the waist by a belt of the same. The sleeves are cut to the elbow, and the cap is also of white linen. The nurses, of course, are never allowed in the wards in this dress.

### Want of Discipline.

One thing struck me very much—namely, the absence of discipline, and the actual disrespect shown by the nurses to the doctors. Fancy an English surgeon going his rounds laughing and joking all the time, and, while he is examining his patient, the nurse lolling on the foot of the bed or in an armchair! Or, again, a house surgeon, supposed to be clean-shaven, going his rounds six days in the week unshaven, and often in his slippers!

### Cooking by Probationers.

The "diet" kitchen is the kitchen in which the special diets are prepared. All the cooking in this department is done by probationers, who are there a month at a time, under the superintendence of the assistant superintendent of nurses. The special diets are much more varied than in an English hospital.

### The Trays.

The doctors do not order any particular article of food—such as a chop, fish, &c.—but a "light tray," "full tray," or "medium tray." On a "light tray" one gets dishes prepared from milk and farinaceous foods, light soups, and, unless expressly forbidden, stewed fruit and ice cream. For a "medium tray" the same, with the addition of fish, poultry, eggs cooked in various ways, toast and butter, milk toast, honey, and various light cakes. The "full tray" comprises all these, with butcher's meat, hot rolls, various kinds of hot bread for breakfast, and raw fruit, such as apples, bananas, water-melon, and other fruits in their season. All meals served in the wards are sent from the kitchens on separate trays of white enamel, the special diets being very daintily served.

### A Kindly Provision.

It is worthy of mention that on application to the medical superintendent at the hospital the patient, if unable to walk, can be fetched by the hospital ambulance, the doctor under whose charge the patient will be always accompanying the ambulance.

### Echoes from the Outside World.

THE number of persons who join the special branch of the Guild of Loyal Women, South Africa, should be very large, because all who have lost friends in the war will naturally feel anxious that the graves of those whom they loved so dearly should not be allowed to go untended, whilst others who have sent forth their kindred to fight for their Queen and country, and have had the happiness of seeing them return unharmed, would wish from motives of thankfulness that the last resting-places of less fortunate soldiers should be preserved as carefully as they would have been had the brave fellows laid down their lives in their own land. Mrs. Stuart, the delegate of the Guild, has received a characteristic letter from the Princess of Wales intimating the satisfaction it gives her to become a patron. Respecting the many who are buried on the veldt, or in Dutch cemeteries, here is a little story of the late Prince Christian Victor, whose affable manner made him so popular with everyone. One day in camp a chaplain to a Highland regiment was anxious to find the Wesleyan chaplain, and as he passed he asked for information from the guard of the tent which was occupied by General Hildyard. The General, recognising the clergyman's voice, invited him in, and pushing forward a seat, they sat and chatted. In the tent was another officer, whose face seemed strangely familiar, although the clergyman was not conscious of ever having met the owner before. When the visitor rose to go, the officer rose too, volunteering to act as guide, because the Wesleyan chaplain's tent was not easy to find. On the way the two conversed pleasantly, and on reaching his destination the clergyman thanked his cicerone, remarking that he had not the pleasure of knowing to whom he was indebted for the courtesy. "Christian Victor" was the quiet reply, and then the Highland chaplain knew that the reason why he had recognised the young officer was because of the striking likeness he bore to the Queen and the Royal Family.

---

IT has been remarked by many how little the public has done to make December 25th a red-letter day for the Tommies in South Africa, who still total up to a large number, and the remark causes one to reflect on the truth of the saying that "gratitude is frequently a sense of favours to come." Last year at this time we felt that the honour of our Empire was at stake, and we despatched shiploads of good fare and gifts to the soldiers who were fighting our battles. This year the Empire again stands firm, and we sit still and congratulate each other that it is so, and no longer bestir ourselves to make plum puddings or to knit socks. Therefore, it is doubly a matter for congratulation that the Secretary of State for War has announced the nation's Christmas box to the soldiers. Every soldier is to be remembered: those of the lowest or fifth class to receive a £5 note each, and of the first class £15 each; and in cases where the soldier has died in warfare or from disease the award will be made to the representatives or relatives. The officers are, of course, not forgotten. Generals—of whom there are only two, Sir Redvers Buller and Lord Kitchener—to receive £2,000 each, and Field Marshals—of whom there is only one, Lord Roberts—to be awarded £2,500. Simultaneously with this announcement comes another which is equally welcome. London is to have the desire of its heart, to welcome "Bobs," and the illustrious victor, accompanied by the Prince of Wales, will repair to St. Paul's for a thanksgiving service on January 3. All details as to route and time have yet to be arranged, but it is confidently believed that the march will be made the occasion of a military display, and it is quite certain it will be an opportunity for the man in the street to give vent to his enthusiasm, which will be eagerly seized.

---

IT is strange that just as we are about to celebrate the home-coming of one famous soldier we should have suffered the loss of some of the most valued relics of a famous sailor. I daresay the fact that a splendid collection of Nelson relics were to be seen in Greenwich Hospital, and were the property of the nation, is unknown to many Londoners, unless they happen to live in the locality; but even these will be moved to indignation and regret that such priceless possessions as the watch and seal worn by Lord Nelson, the various medals given to the celebrated commander, and the bullet which killed him, should have been made away with. The difficulty of discovering an adequate motive for such a robbery—for it would be almost impossible to dispose of such well-known treasures till at least a number of years had elapsed—has given rise to the rumour that three suspicious Frenchmen were seen to be hovering about on Friday and Saturday, and that they have now disappeared. It is quite probable that the Frenchmen are not the culprits, but in the absence of any direct statement from the police or the Greenwich Hospital authorities, it is natural to attribute the outrage to a foreign political agency.

---

THE announcement that the Duke of Westminster is engaged to be married to Miss Shelagh Cornwallis West is unusually interesting, not only because the prospective bridegroom bears such an honoured name, and has so lately returned from the front, but also because it is such a short time since the prospective bride's brother was united to Lady Randolph Churchill. Miss West is a great beauty, like her sister, Princess Henry of Pless, and she has lately been staying with her grandmother, Lady Olivia Fitzpatrick, at The Warren, near Chester. This gave the young couple the opportunity of meeting, and apparently an old story was enacted again, for, like Desdemona, "she loved him for the dangers he had passed." But the Duke and Miss West have known each other since they were children, and their engagement is a direct refutation of the idea that old friends seldom marry. In the Grosvenor family in particular the men are evidently especially attracted by those whom they have known for years, and whose truth and sterling good qualities they have had opportunities of proving for themselves. The late Duke of Westminster married for his second wife a lady whom he had known when she was in the schoolroom, and whom he had seen develop year by year; and now his grandson follows in his steps. The late Duke's marriage, it is well known, was a particularly happy one. May the present Duke be equally fortunate!

---

OFTEN it falls to the lot of nurses who are connected with public institutions to take part in a bazaar, a fête, or some similar function designed to assist the funds of the establishment where they work. The regular stalls are generally easily organised, but it is difficult to arrange for anything new in the way of a side show. The Art Gallery, consisting of genuine pictures or of a comical travesty of the real thing; the Curiosity Show, the Palmistry Tent, or the Waxwork groups are all well known; but the Nail-driving Competition is, I think, somewhat of a novelty, and decidedly it is cheeringly remunerative to the gentleman in charge. All that is required is a small room or a space curtained off a large hall, a piece of thick wood—or two if many ladies and children are likely to compete—a firm block or table upon which to place the wood, a hammer, and some long, strong nails. The object of the contest is to find who can drive the nail the farthest into the wood. The nail may be held whilst the first tap is given, as it is supposed only to settle it sufficiently to cause it to stand alone, but the next two blows must be dealt without the hand touching the nail. Thus, three strokes in all may be struck. A little piece of cardboard bearing the name of the person is run on to each nail before it is handed to the competitor, and it is better to keep the ladies' attempts and gentlemen's distinct, as naturally a woman is at a disadvantage when competing with a man, in matters requiring strength and practice. Twopence is the general charge for each trial, and experience shows that many people desirous of succeeding return again and again to try their skill. It is usual to offer three prizes—one for men, one for women and one for those under fifteen.

### Everybody's Opinion.

### THE NURSING OF ENTERIC.

Mr. G. W. Ord, M.R.C.S., writes from 58 Queen's Road, Richmond: I shall be glad to hear from any nurse who has contracted enteric while nursing an enteric case. A postcard stating the length, in days, of her fever to the day when the temperature first became normal will afford the information required.

### STERILISING GLOVES IN CANADIAN HOSPITALS.

A Canadian Nurse writes: Thinking it might possibly interest some of your readers, I venture to send you the method of sterilising gloves as employed in one of the best Canadian hospitals. The gloves were carefully wrapped in double gauze inside out, then boiled in distilled water between two basins for 15 minutes, the water drained away, the upper basin still covering the gloves unless wanted at once. The gloves were then carefully wiped by a nurse with sterile hands and a sterile towel, turned and dusted with sterile talcum powder inside, when they were ready for the surgeon, the process enabling the surgeon to have his hands dry inside the gloves.

### NOVEL USE FOR A DISTRICT NURSE.

"A Trained Nurse" writes: The novel use for the district nurse is, alas! not so novel as one would wish. But the would-be patient is not always to blame. Ladies visiting the poor and hearing a list of troubles, of which sickness is by no means the chief, though it is included, often say, "Call in the nurse, she is very nice, and will put you right, &c.," leaving often a mistaken idea of a nurse's duty. Again, some committees require eight hours' duty daily. Should the place be in a healthy condition or should there be a rush of bad cases the nurse is reprimanded for either not working her time or exceeding it. In one case I was reprimanded for going to a case at 11 P.M., where, had I not gone, death would have occurred. What I fear is the great temptation to young nurses to fill up the time and so blot out true sympathy which their sisters so often try to train in them. I fear the respect for nurses is going down, and will continue to do so until a more strict limit is made as to who shall and who shall not wear uniform.

### NURSING IN THE EAST.

"Colonial" writes: In the interests of the nursing profession at large, I would warn all nurses intending to join institutions or hospitals in the East to make stringent inquiries respecting diet, quality as well as quantity. Get a list of all the articles of food supplied. Make yourselves acquainted with the relative value of colonial monies and the prices of the necessities of life. You cannot do housework and cooking in a tropical climate, even for yourself only. See that servants are provided for you; also some sort of conveyance, as often it is impossible to walk on account of the heat and the dust. These "little things" make big holes in small purses. Employers are apt to romance about the advantages, and to ignore the disadvantages, of the posts they offer their employés. It is advisable to write to some disinterested member of the institution you intend joining for "facts"—bare and truthful. All, or nearly all, who join colonial institutions from England are bound to sign an agreement for a certain time. In the event of their wishing to leave during that period, they have to refund passage-money or forfeit salary. Take the advice of one who has been "done," and "look before you leap."

### RULES IN PRIVATE NURSING.

"A Watcher" writes: I should be so grateful if some fellow-nurses would kindly inform me what is their rule in private nursing with regard to the giving of medicine when the patient is asleep. Supposing he sleeps an hour or more beyond the time the (say 4-hourly) dose is due, is the time counted from the moment the dose is taken? This involves the constant noting in writing of the hour the medicine is due, and if one cannot instantly note it down one is apt to forget the exact time. Then, if one follows this plan, probably half a dose or more is lost in the twenty-four hours. On the other hand, if one adheres to fixed hours two doses may come very near together. And in the case of *two* medicines the difficulty is doubled. I should also be glad to know if it is usual for a nurse to sleep in a convalescent *man's* room, i.e., when he only needs attention once or twice in a night. I heard a matron of a nursing institute refer incidentally to this practice as if it were a matter of course. To me the idea seems utterly opposed to all notions of modesty, and I feel that I would rather lose a case than agree to do so. Is it generally expected, and of *young* nurses?

### THE MADRAS GENERAL HOSPITAL.

"An Englishwoman who Trained There" writes I think "Another who Knows the Hospital" knows really very littleabout it. The English ladies who went to train there may in their turn say, "The matron is Irish and the doctors are Britishers, but the right sort do not find their way out here, as probably it is easier to get work in a better climate." Doubtless much has been done to raise the standard of the hospital, but much remains to be done. I have not known English nurses to complain of the hospital, its conveniences or arrangements for work, &c., but of those who direct it; they do not care particularly for "what *used* to be;" it is the private treatment they get at the hands of those in authority that affects them personally and makes or mars their happiness. It is wrong to say they expect more pay, *no* restrictions, and a minimum of work. A form is usually given to the would-be nurse to fill up before joining. I think it states the hours of work and pay; but I regret to say that all the rules concerning the nurses are not on the form, and it reads pleasanter than the reality is. No nurse expects much pay when she is training; as for restrictions there must be some; but an Englishwoman likes fairness, so let one rule stand for all nurses. When the matron was installed she may have been "entirely subordinate to the senior medical officer," but with the lapse of years that order has changed.

### MOSQUITOES AND ENTERIC FEVER.

"A Sufferer" writes: I read with great interest the reference recently made in your columns to the article by Dr. Howard Tooth, which appeared in the *British Medical Journal*, where he enumerated the various modes by which the infection of enteric fever was spread in South Africa. Bearing in mind the experimental work which is being carried out in the Roman Campagna, it may interest you to hear of the conclusions at which I had arrived from my own experience of mosquitoes with regard to enteric fever previous to my hearing anything of the theory now being tested by Dr. Sambon and Dr. Low, and I may say before they went out. I am a member of the Army Nursing Service Reserve, and was sent to South Africa in February last. I proceeded to Bloemfontein by the first train after its surrender: there I noticed that when at work in the enteric wards the mosquito bites, from which we—the nursing sisters—suffered considerably were unusually inflamed, swollen, and irritable. After three weeks I was removed to the surgical division, where, to my surprise, I found the only result from the bite was a minute red spot. Later on, returning to enteric work, the virulent aspect reappeared, which caused me to express to several my conviction that we were literally being inoculated with enteric by *mosquitoes*, and I very shortly fell a victim to this terrible malady. Dr. Tooth himself visited me several times in consultation during my illness. Of course, I am quite aware that many people suffer a good deal of inconvenience from the bites of these insects, and I saw several cruelly disfigured even before they started work; but the bite of what I call the infected mosquito appears to me to be of a totally different nature. I should very much like to know the opinion of others on this subject, especially those who have been through the present campaign.

[It must be remembered that any such process of infection by mosquitoes as is here suggested must be placed in quite a different category from that by which malaria is transferred from case to case, the malaria organism not being merely carried by the mosquito, but undergoing one phase of its life-cycle within the body of that insect.—Ed. Hospital.]

### A Book and its Story.

MRS. HUMPHRY WARD'S NEW NOVEL "ELEANOR."

By Mrs. Humphry Ward. (Smith, Elder & Co., 6s.)

Although Mrs. Humphry Ward has named her new novel "Eleanor," it is only subjectively that her character claims the attention and interest of the reader. For with her fated shadowed life, her delicate sensitive temperament, with an intangible personal beauty, having its origin rather within than without, of that rare and complex order which it is given to a few to recognise and fewer appreciate, she stands a pathetic figure in the background, an inspiration and support to other characters. Her own sorrows have made her sympathetic. At thirty she is practically alone in the world, with a life to re-make; a disenchanted woman, married early to a husband who has failed to make her happy. Losing both him and her child by one tragic stroke, she found a sanctuary with a maiden aunt, Miss Manisty, and her nephew Edward. He was in the prime of life, unmarried, heir to a property, and had succeeded his father as Member for the Division he had represented in the Liberal interest. But "Manisty had been abroad for seven or eight years, living with all the bigwigs and reactionaries everywhere. The last thing in the world he knew anything about was English politics." He had, naturally, succeeded his father, but his restless, eager mind, with its admiration for everything historically interesting, matured by time and tradition, made him unfit for his father's school of politics. "He was not meant to be a Liberal," nor a politician at all, perhaps. He accepted his position tacitly at first, but before very long he had offended his party, and, at the time the story opens, had come to Italy "to escape the frictions and agitations which his actions had brought upon him." Here, in an old Italian villa he had established himself with his aunt and cousin Eleanor. The old-world charm pervading the place delighted him. High up on a ridge of the Alban Hills it stood, with slope after slope shelving down, bearing vineyards and pine plantations on its terraced surface, till it touched the Campagna. To the north, beyond the plain, lay Rome.

In this secluded retreat Manisty intended to bring out a book. "He had previously written brilliantly, and he was bent upon startling the world with his latest utterances on Modern Italy as an object lesson to England. It was a hostile and passionate study of Italy, the new country made by revolution, fashioned, so far as laws and government can do it, by the lay modern spirit. In reality the book was a party pamphlet, written by Manisty in defence of certain acts which made him for a time the scandal and offence of the English political party in whose interests he had entered Parliament and taken office." With this book, doomed from the outset to failure, his cousin Eleanor was assisting, acting as his secretary, patiently loyal, working indefatigably by his side, even when weary and out of sympathy with its object. But by thus sacrificing herself voluntarily to Manisty's interest in his work, she hoped vaguely to regain some interest in life. In it she found a new reason to live. "How strange that he and she should be engaged in this work together, this impassioned defence of tradition, of Catholicism and the Papacy, as the unperishable, undestructible things. . . . . He, one of the most thorough sceptics of his day, as she had good reason to know, she, a woman who had at one time ceased to believe, because of an intolerable anguish, and was now only creeping slowly back to faith, to hope. . . . . The even tenor of their daily routine is disturbed before long by the announcement of the expected visit of a little New England girl, whose relative in Boston had hospitably received Miss Manisty and her nephew when in the States. "Such luncheons and dinners!" Miss Manisty raised her gouty little hands. "My dear, when we left Boston I never wanted to eat again. It would be simply indecent if we did nothing for this girl. English people are so ungrateful this side of the water. It makes me hot when I think of all they did for us." By a series of accidents Lucy Foster's visit has been unavoidably hastened. One friend after another, into whose care she has been given, have from illness, and other causes, failed. And the news of her sudden advent was not amicably received by Manisty. The book was developing; everything he decided depended on close, uninterrupted application on his and Eleanor's part at this juncture, if it was to be brought to a satisfactory—from his point of view—conclusion. One morning the post brought a letter from Lucy Foster saying she would be with them later in the day. Although Lucy's visit was the outcome of an invitation from Miss Manisty dictated by her nephew, he, with characteristic masculine obliviousness to so small a detail, exclaimed, "Let us be quite clear, Aunt Pattie—when does this young woman arrive?" "In about half an hour. But really, Edward, you need take no trouble; neither you nor Eleanor need trouble your heads about her." "One never prepares for these catastrophes until they actually arrive," Manisty muttered: "How is one to be civil about this visit? Nothing could be more unfortunate. These last critical weeks, and each of us so dependent upon the other. Really it is the most monstrous folly on all our parts that we should have brought this girl upon us." The keynote of Manisty's character was contradiction. Not very far on, a few days following the arrival of Lucy Foster, "whose grandfather was a divinity professor, and wrote a book on the Inquisition." Lucy, who, as Eleanor was quick to descern, possessed a curious likeness to a certain little statuette head of Artemis which stood on the mantelpiece of the Salon, was already beginning to excite interest in his preoccupied mind. Perhaps it was this which caused him to regard her with critical attention one day as she crossed his vision, when, for some subtle reason, not certainly connected with "Modern Italy," his mind was running on "women—their delightfulness—and his own inclination for their society." . . . . And here came this girl walking through his dream to remind him of what "woman," the average virtuous woman of the old or new world is really like. "Manisty's collective noun referred merely to that small, high-bred cosmopolitan class which presents types like Eleanor Burgoyne. All the same, Lucy Foster walked well—she carried her head remarkably well There was a free-springing youth in all her movements that he could not but follow with eyes that noticed all such things as she passed through the old trees and the fragments of Græco-Roman sculpture placed among them." From the coming of Lucy, entering all unconsciously into the intellectual exclusive atmosphere of the villa, Eleanor intuitively perceives the inevitable consequence. On her side Manisty was filling up the unoccupied spaces of a heart torn too early—by doubt and bereavement—but she could not close her eyes to the possible effect of Lucy's youthful charm to a mind already satiated with a world whose women were otherwise than this simple little New England girl, strong with a rigid moral strength and directness, inherited from Puritan ancestry. It is in the working out of the denouement that Mrs. Humphry Ward has put all the force of her analytical faculty. Eleanor Burgoyne's life began in disillusionment, and ends in self-renunciation. The delineation of these two women is a touching and masterly one. More than once one is reminded in reading of Manisty and his relations to Lucy, especially after the introduction of his poor mad sister, of an imperishable work of fiction written in the early Victorian days, and still read with avidity, as new editions testify. But we doubt if "Eleanor" will forty years hence find a public, for while the former book appeals to human nature perennially, "Eleanor's" public will be a smaller, and necessarily more evanescent one, but by them will be read and appreciated.

### For Reading to the Sick.

TRIBULATION worketh patience; and patience, experience; and experience, hope.—*Rom.* v. 3-4.

Called aside;—
O, knowledge deeper grows with Him alone,
In secret oft His deeper love is shown,
And learnt in many an hour of dark distress
Some rare, sweet lesson of His tenderness.

Called aside;—
We thank Thee for the stillness and the shade;
We thank Thee for the hidden paths Thy love hath made,
And so that we have wept and watched with Thee,
We thank Thee for our dark Gethsemane.

Called aside;—
O restful thought,—He doeth all things well,
O, blessed sense, with Christ alone to dwell:
So, in the shadow of Thy Cross to hide,
We thank Thee, Lord, to have been called aside.—*Anon.*

So much has been said and written about meditation, and yet, I suppose, most of us still have our own peculiar difficulties and troubles concerning it. All spiritual writers agree that it is a necessity, indispensable to the life of growth in holiness. But it must vary in many respects, according to the capacities and dispositions of different people; and while systems and minute plans help some, they do but hinder and perplex others. We very often feel in meditation that our heart is as cold as a stone. Then there are times when one is so restless and distracted that it seems impossible to fix one's mind on anything. If the weakness caused by illness distracts you, say, "Lord, he whom Thou lovest is sick." No subject will so help the sick as our Lord's Passion. It is often well at such a time to take a few words from the Bible, a single verse, perhaps, or to go on reading some narrative until something fixes the attention and kindles the affections. But if even this fails, and one feels perfectly empty and dry, we must wait on humbly, ask God to speak when He will to the soul, and be patient. The mere making such an act of humility, and of waiting upon God, is a gain. Trying to be calm, place yourself in God's presence; ask Him for strength if you are weak, or for gentleness if you are over-active. If even a book wearies you, put it down; accept your incapacity humbly, and be content with using ejaculations, or an "Our Father" devoutly said.—*Sidney Lear.*

Oh, when my hour is come, if so Thou wilt,
Let the sweet blossoms of the bough of love
Hang o'er my bed. But, howsoe'er it be,
Thro' the night watches, till the birds awake
Their sad importunate music, till the morn
Pale on the pane, oh, let me wait for God!
Gently, my Saviour! stand beside the door;
Gently, my Saviour! through the lattice glide;
Dip my life's leaves, adust with thought and care,
In sacramental dews, and make them gold.
Rest over me in love, O piercèd One!
Smile on me sadly through my mist of sin,
Smile on me sweetly from Thy crown of thorns.
As the dawn looketh on the great dark hills,
As the hills dawn-touch'd on the great dark sea,
Dawn on my heart's great darkness, Prince of peace!

*Bishop Alexander.*

### Where to Go.

MONDAY, DECEMBER 17.—Pianoforte and vocal recital on behalf of the Homes for Working Boys in London at the Bishopsgate Institute at 8 o'clock, by Mr. Geo. Denham, assisted by Mrs. Chambers, Madame Amy Young, &c., &c.

### Notes and Queries.

**The Editor is always willing to answer in this column, without any fee, all reasonable questions, as soon as possible.**
**But the following rules must be carefully observed:—**
**1. Every communication must be accompanied by the name and address of the writer.**
**2. The question must always bear upon nursing, directly or indirectly.**
**If an answer is required by letter a fee of half-a-crown must be enclosed with the note containing the inquiry.**

*Open-air Nursing.*

(107) How can I get a list of open-air sanatoria? Is it necessary to receive training in general nursing before entering one as nurse; and is there much lifting? I have had some training in an institution for sick children, but as I am not strong enough for regular hospital work, I have been advised to try open-air work.—*Ruth H.*

Apply to the publishers of the "West London Medical Journal," 89 Great Titchfield Street, W.

*Invalid Appliances.*

(108) Can you tell me of any kind of apparatus for keeping a patient from slipping down in bed?—*C. J.*

See the advertisements of makers of invalid appliances in our advertisement columns; you will find several to choose from.

*Testing Urine.*

(109) Would the Editor kindly tell Nurse G. the best and quickest way of testing urine for (*a*) albumen and for (*b*) blood?

(*a*) Place some strong nitric acid in a test tube and pour some urine gently down the side of the tube. A white band of albumen will appear at the junction of the two liquids. (*b*) Add to the urine in the test tube a few drops of tincture of guaiacum, and shake violently. Then add about half an inch of ozonic ether, and, if blood be present, a blue colour will form on the uppermost layer.

*Book.*

(110) I am attending lectures on "First Aid." What text-book do you advise me to use?—*Probationer.*

You must, of course, use the book recommended by your lecturer.

*Comparison.*

(111) Will you kindly tell me the difference between a paying probationer and one who receives a salary? Is a paying probationer better taught, and are her duties lighter?—*A. M.*

The advantage, if there be any difference in the treatment of the two, lies nowadays with the salaried nurse. The duties and instruction of both are nearly, if not altogether, identical; and, whilst the salaried nurse is paid, the paying probationer pays.

*Home for Lady.*

(112) Can you tell me of a home for an old lady of 74, at from 4s. to 5s. a week? She is not bedridden, but suffers from indigestion and a weak heart. —*Nurse T.*

There are a few homes which receive such cases from 7s. a week. If this is too much the only thing to be done is to try and get her admitted to the Royal Hospital for Incurables. Address the Secretary, 106 Queen Victoria Street, E.C.

*Non-Alcoholic.*

(113) Is there a hospital in London at which no alcohol is given to the patients? If so, would you kindly tell me which one?—*M. McI.*

The London Temperance Hospital, Hampstead Road, N.W.

*Visiting Nurse.*

(114) 1. I am thinking of beginning work as a visiting nurse. Is the charge made for the hour or for the visit? Is 10s. 6d. too much for attending an emergency confinement, or for attending an operation? 2. Would it be unprofessional to advertise in local papers?—*Policy* 3523.

1. The "Note" entitled "A Visiting Nurses' Association" in the MIRROR for October 20th answers your questions fully. 2. A nurse may advertise where she thinks fit.

*Medical Adviser.*

(115) My friends wish me to have a good medical opinion, but I do not know to whom to go. Would you kindly tell me of a general physician whom I could consult in London?—*M. N.*

We cannot recommend individual practitioners. If, however, you require to consult a specialist, choose one of the physicians on the staff of any of the hospitals devoted to the class of disorder from which you suffer.

*Infirmary Training.*

(116) Does your answer to Louise P. (No. 272 in the MIRROR of September 29th) include large workhouse infirmaries or hospitals under the Local Government Board?—*Ignoramus.*

Certainly; but you must remember that it is good methods and good teachers that make a good school. not the building that accommodates them.

**Standard Books of Reference.**

"The Nursing Profession: How and Where to Train." 2s. net; post free, 2s. 4d.
"The Nurses' Dictionary of Medical Terms." 2s.
"Burdett's Series of Nursing Text-Books." 1s. each.
"A Handbook for Nurses." (Illustrated.) 5s.
"Nursing: Its Theory and Practice." New Edition. 3s. 6d.
"Helps in Sickness and to Health." Fifteenth Thousand.
"The Physiological Feeding of Infants." 1s.
"The Physiological Nursery Chart." 1s.; post free. 1s. 3d.
"Hospital Expenditure: The Commissariat." 2s. 6d.

All these are published by THE SCIENTIFIC PRESS, Ltd., and may be obtained through any bookseller or direct from the publishers, 28 and 29 Southampton Street, London, W.C.

### Travel Notes.

BY OUR TRAVELLING CORRESPONDENT.

### LXIII.—SIX MONTHS IN ROME.

THE beautiful city is terribly spoilt by the greedy hands of speculators and builders. I am told that thirty years ago, before Rome became a Royal capital, it was infinitely more interesting; but it is not, I fancy, Royal occupation that has had so fatal an effect, nor as devout ultra-montanes believe the decline of the Papal secular power, but rather the greed of Romans themselves, who, dreaming of making their city a huge modern Royal capital, and reimbursing themselves largely, squandered vast sums in destroying many a beautifu relic of the past and erecting acres of frightful houses which remain an unoccupied eyesore. To effect this atrocity the charming old streets have been torn down, and it is really some comfort to hear that many speculative builders have been ruined in the process. A very excellent and impartial account of this building mania may be found in Crawford's "Don Orsino."

### ST. PETER'S AND THE PIAZZA.

These remain untouched, and I hastened there very early in my stay. It is well to enter the Piazza by going through the Piazza Rusticucci in remembrance of Raffaelle, who died there under the shadow of his immortal works in the Vatican, and lay in state with his last and (generally considered) greatest work, "The Transfiguration," hung over his bed. In the centre of the Piazza di San Pietro stands the famous obelisk from Heliopolis. Formerly it was near the present sacristy, but in 1586 Sextus V. brought it on rollers to the spot it now occupies. It was not erected without peril and difficulty; silence was imposed on the waiting multitude under pain of death, and the obelisk slowly rose towards its position; but the engineer had not rightly judged the huge weight, the ropes began to give under the strain when a sailor of Bordighera in defiance of the command shouted "Water on the ropes!" The situation was saved, and the privilege of providing palms for St. Peter's granted to the inhabitants of Bordighera in perpetuity. This magnificent Piazza was desecrated to the uses of a bull-ring in 1500 by Cesare Borgia, son of the infamous Alexander VI.

### THE INTERIOR OF ST. PETER'S.

The first feeling on entering St. Peter's is one of coldness, due to the absence of the rich coloured glass we are accustomed to in our Northern churches; this wears off, however, and one is impressed with the magnificence of the whole. You will find yourself often in St. Peter's, and there is, indeed, much to see there. A pathetic interest attaches to the graves of our own exiled Stuarts. Here lies the fanatic James II. and his son called the "old Pretender." Here, too, lie the two sons of the latter, Charles Edward and Henry Cardinal York. Of Charles Edward, that romantic and charming personality, one must ever regret that the close of his embittered life was so sadly at variance with his chivalrous early years. However much we must mourn the latter days of that stormy life, we must remember he was once gallant, brave and true, and to the last retained the devoted affection of thousands. Peace to his ashes in their splendid resting-place.

### THE CRYPT OF ST. PETER'S.

This is one of the most interesting parts of the mighty church and can only be seen by an order obtained from the Palazzo della Cancellaria. It is all that remains of the old Basilica erected on the circus of Nero to commemorate the martyrdom of St. Peter and other Christians who suffered on this spot. Here are buried many Popes prior to the middle of the sixteenth century, among others the Borgia Pope, Alexander VI., but his body, abhorred by all, was removed by order of Julius II., his successor, and placed in another church, since destroyed. Eventually his bones were allowed a permanent resting place in S. Maria di Monserrato. I shall have more to say of this hated but luridly-interesting family later on. In the church above you will see the celebrated figure of St. Peter, popularly supposed to have been cast from a statue of Jupiter. Thousands of lips have kissed the extended toe and hoped to gain therefrom temporal and spiritual blessings. What magnificent scenes both the exterior and interior of St. Peter's have witnessed! What a moment of burning interest that must have been when, in 1846, Giovanni Mastai gave out from the balcony to the waiting and breathless multitude the announcement of his election to the Pontifical throne, which he ascended as Pio Nono! It was the last time that a Pope was elected with regal as well as ecclesiastical honours, for long ere Leo XIII. ascended the Pontifical chair the Papal States existed no longer. A national desire for freedom and a more liberal Government, and a hope that in a united Italy healing might be found for internal wounds, brought about through a variety of secondary causes the abolition of the Papal temporal power and united the country under a Piedmontese King. In 1847 the troubles began. There were faults on both sides. Rome is still divided into two parties on this burning question between Pope and King. The white, or royalist party, many of them devout Catholics, would fain be friends with the black; but the black, or Pontifical party, will make no terms with the rebels, as they consider them, and the oppressors of the prisoner of the Vatican, as they somewhat absurdly call His Holiness. If you live in Rome and mix in Roman society, you can steer no middle course—black or white you *must* be—no grey tints are admissible. Pio Nono was a good but a weak man; his life was of blameless purity and full of deeds of mercy and kindness, but he was incapable of commanding the difficult situation in which he found himself, and a sinister adviser was always at his elbow in the astute Cardinal Antonelli.

### BOOKS ON ROME TO STUDY.

The pleasure of a visit to Rome is greatly enhanced by previous study. The following books may help you considerably:—Hare's *Walks in Rome*; this is excellent, but deals more with ancient than mediæval Rome. *Roman Gossip*, by Mrs. Elliot; this is in a much lighter vein, as you may suppose, but contains much information, acquired on the spot, of the last 50 years of Roman history. Ouida's *Ariadne* is most charming and picturesque, and Hawthorn's *Transformation*, which is known to most English and American readers. Amongst rather rarer and deeper books, in a sense, one may put Vasari's *Lives of the Painters*, Kugler's *Italian Schools of Painting*, Heman's *History of Mediæval Christianity and Sacred Art*, and Lanciani's *Pagan and Christian Rome.*

---

### TRAVEL NOTES AND QUERIES.

RULES IN REGARD TO CORRESPONDENCE FOR THIS SECTION.—All questioners must use a pseudonym for publication, but the communication must also bear the writer's own name and address as well, which will be regarded as confidential. All such communications to be addressed "Travel Editor, 'Nursing Mirror,' 28 Southampton Street, Strand." No charge will be made for inserting and answering questions in the inquiry column, and all will be answered in rotation as space permits. If an answer by letter is required, a stamped and addressed envelope must be enclosed, together with 2s. 6d., which fee will be devoted to the objects of the "'Hospital' Convalescent Fund." Any inquiries reaching the office after Monday cannot be answered in "The Mirror" of the current week."

PEGLI (Anxious One).—It is a charming place if (a large if) your friend is able to get about on excursions, otherwise I should not recommend it: too small and confined, but it is in constant communication with Genoa by rail, and is near to innumerable lovely spots. You might ask for terms at the Grand Hotël. Its chief attraction to most tourists is its ridiculous Villa Pallavicini, a place arranged in the worst possible style.

ST. JACUT (Artist).—St. Jacut is in the Côtes-du-Nord. There is still a convent there that takes guests, but it is not particularly cheap, and it has become now quite a little English centre in the summer season. It is not fitted to meet your requirements. You must go to lower Brittany to find costume and primitive habits, and to a small place, such as Audierne (on the line), or better still, Penmarc'h, much more secluded. From either you can study the magnificent coast round the Baie des Trépassés.

BRITTANY FOR THREE MONTHS (Alex).—Your plan is excellent, as you are not tied to season; by all means take May, June and July. Start from St. Malo, strike south-west by St. Brieuc and Pontivy to the country round Auray, taking in Vannes and the Morbihan and working up slowly round the coast to Quimper, Baie des Trépassés and Le Faouet; from there turn inland to the country of the Montagnes Noires, up by Huelgoat and Morlaix. From there work all round the coast to St. Malo again. Expenses need never exceed 6s. 8d. per day, and will often be much less.

## Figures and Tables

**Figure f1:**